# Supplementary material for: Development of the ECLIPSE model of meaningful outcome domains following lower limb amputation and prosthetic rehabilitation, through systematic review and best fit framework synthesis
Source: PLoS One. 2024 Jul 23;19(7):e0307523. doi: 10.1371/journal.pone.0307523 (PMC11265722; doi:10.1371/journal.pone.0307523)
Supplement: S1 Appendix — The Authors previously developed conceptual model of outcome domains of importance following lower limb prosthetic rehabilitation. (DOCX) [file pone.0307523.s002.docx]

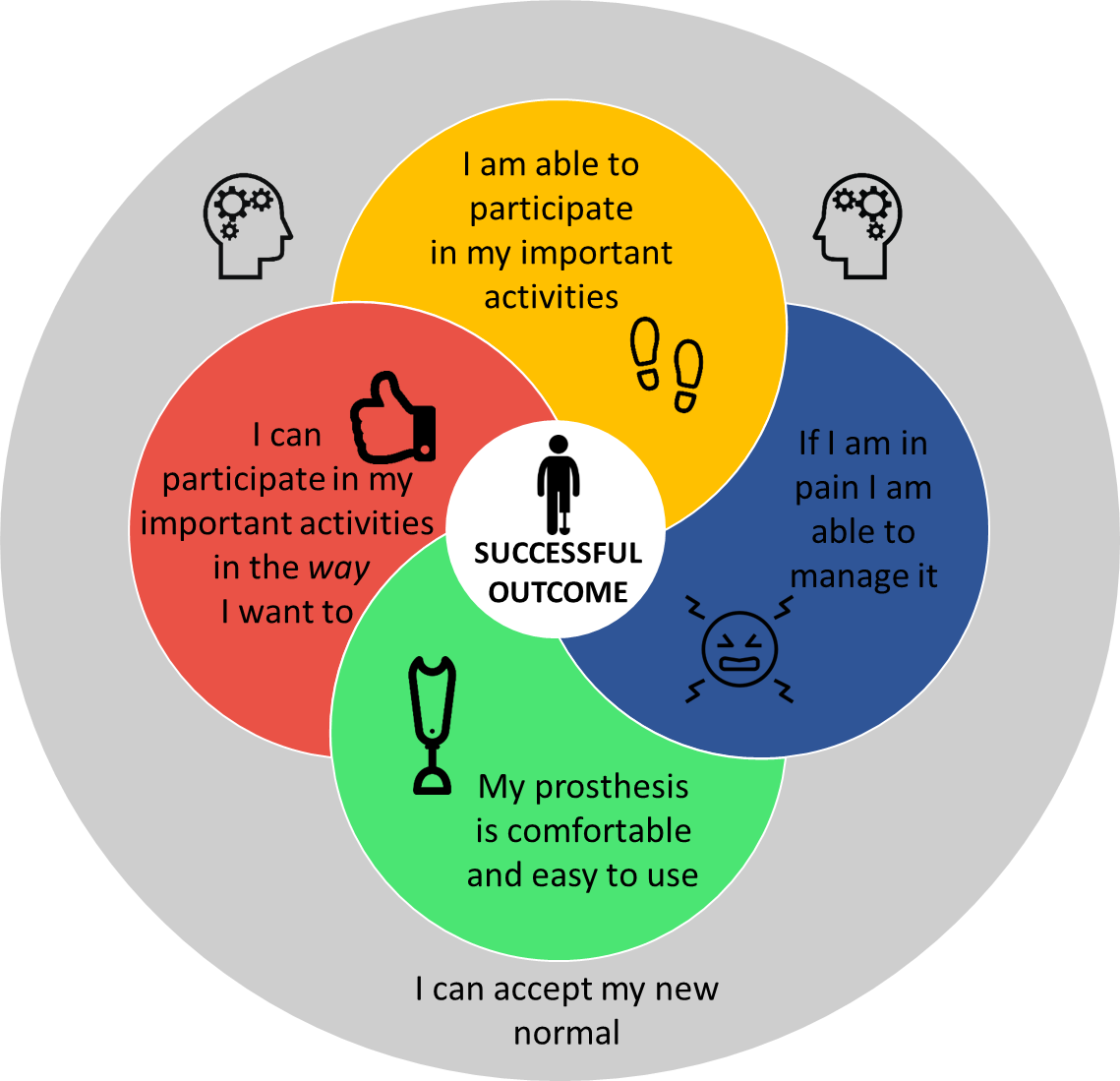


**Figure in S1 Appendix. Pre-existing conceptual model.** The Authors previously developed conceptual model of outcome domains of importance following lower limb prosthetic rehabilitation. Reprinted from Ostler C, Donovan-Hall M, Dickinson A, Metcalf C. Exploring meaningful outcome domains of recovery following lower limb amputation and prosthetic rehabilitation: the patient’s perspective. Disabil Rehabil. 2022;0: 1–14. doi:10.1080/09638288.2022.2138989 under a CC BY license, with permission from Taylor and Francis, original copyright 2022.
