# Supplementary material for: Development of the ECLIPSE model of meaningful outcome domains following lower limb amputation and prosthetic rehabilitation, through systematic review and best fit framework synthesis
Source: PLoS One. 2024 Jul 23;19(7):e0307523. doi: 10.1371/journal.pone.0307523 (PMC11265722; doi:10.1371/journal.pone.0307523)
Supplement: Attachment — Submitted filename: Reviewers response letter.docx [file pone.0307523.s005.docx]

Dear Sir or Madam,

Portsmouth Enablement Centre

St Marys Community Health Campus

Milton Road

Portsmouth

PO3 6AD

Tel: 07843 283147

Email: chantel.ostler2@porthosp.nhs.uk

**19/12/2023**

**Re: Resubmission of a previous article (PONE-D-23-40224) A qualitative synthesis titled `Development of the ECLIPSE model of meaningful outcome domains following lower limb amputation and prosthetic rehabilitation, through systematic review and `Best Fit’ framework synthesis.’**

Please see below a table with a response to the editor’s comments.

| Comment | Response |
| --- | --- |
| In the case of your submission, we are concerned that the literature review was completed in 2022. Systematic and scoping reviews should provide an up-to-date overview of the research topic covered. Since the literature search was completed over a year ago, we are concerned that this study does not meet this requirement.  As a result of these concerns, we cannot consider the manuscript for publication at PLOS ONE at this time. However, if you are able to address the concerns discussed above, we may be able to consider a revised version of the manuscript. This would however require a substantial amount of work and thus, would be considered as a new submission. If you are able to revise the manuscript to respond to the concerns noted above, please refer to the original submission in the covering letter and accompany the submission by a rebuttal letter that responds to the comments made on the original manuscript. | This review was carried out in early 2023. Unfortunately, there was an error in the original manuscript which stated early 2022 which may have led to your decision that the review was undertaken too long ago. As the work was actually done within the past year we ask that you reconsider your position. We have now corrected the date within the article.  We apologise profusely for this error which should have been picked up during preparation of the manuscript. Our statement of error is supported by table 6 in the main manuscript which lists the papers included in the review. Many papers from throughout 2022 where included.  Again we offer our sincere apologies and hope you will reconsider our submission. We also apologise for any time wasted. |

Thank you for reconsidering this manuscript and we look forward to receiving your thoughts.

Yours Sincerely

Chantel Ostler

Corresponding author

Consultant Clinical Academic Physiotherapist – Prosthetic Rehabilitation
