# Supplementary material for: Development of the ECLIPSE model of meaningful outcome domains following lower limb amputation and prosthetic rehabilitation, through systematic review and best fit framework synthesis
Source: PLoS One. 2024 Jul 23;19(7):e0307523. doi: 10.1371/journal.pone.0307523 (PMC11265722; doi:10.1371/journal.pone.0307523)
Supplement: Attachment — Submitted filename: Response to Reviewers.docx [file pone.0307523.s006.docx]

**Response to reviewers’ table**

| **Reference point** | **Comment** | **Response** |
| --- | --- | --- |
| 1. | Please ensure that your manuscript meets PLOS ONE's style requirements, including those for file naming. | Completed as requested |
| 2. | Please include this amended Role of Funder statement in your cover letter; we will change the online submission form on your behalf. | Included above |
| 3. | We note that Figure 1 in your submission contain copyrighted images. All PLOS content is published under the Creative Commons Attribution License (CC BY 4.0), which means that the manuscript, images, and Supporting Information files will be freely available online, and any third party is permitted to access, download, copy, distribute, and use these materials in any way, even commercially, with proper attribution. For more information, see our copyright guidelines: [http://journals.plos.org/plosone/s/licenses-and-copyright](https://eur03.safelinks.protection.outlook.com/?url=http%3A%2F%2Fjournals.plos.org%2Fplosone%2Fs%2Flicenses-and-copyright&data=05%7C02%7CC.Ostler%40soton.ac.uk%7C7cad507318504b07d1d808dc703e0007%7C4a5378f929f44d3ebe89669d03ada9d8%7C0%7C0%7C638508658790332391%7CUnknown%7CTWFpbGZsb3d8eyJWIjoiMC4wLjAwMDAiLCJQIjoiV2luMzIiLCJBTiI6Ik1haWwiLCJXVCI6Mn0%3D%7C0%7C%7C%7C&sdata=bUCbn55B4qFrWDPjnLOOF4uf%2FCtAJ62E7xUMv4da6AQ%3D&reserved=0). We require you to either (1) present written permission from the copyright holder to publish these figures specifically under the CC BY 4.0 license, or (2) remove the figures from your submission: a. You may seek permission from the original copyright holder of Figure 1 to publish the content specifically under the CC BY 4.0 license.  We recommend that you contact the original copyright holder with the Content Permission Form ([http://journals.plos.org/plosone/s/file?id=7c09/content-permission-form.pdf](https://eur03.safelinks.protection.outlook.com/?url=http%3A%2F%2Fjournals.plos.org%2Fplosone%2Fs%2Ffile%3Fid%3D7c09%2Fcontent-permission-form.pdf&data=05%7C02%7CC.Ostler%40soton.ac.uk%7C7cad507318504b07d1d808dc703e0007%7C4a5378f929f44d3ebe89669d03ada9d8%7C0%7C0%7C638508658790339271%7CUnknown%7CTWFpbGZsb3d8eyJWIjoiMC4wLjAwMDAiLCJQIjoiV2luMzIiLCJBTiI6Ik1haWwiLCJXVCI6Mn0%3D%7C0%7C%7C%7C&sdata=5TZkMUNMXtOqX%2F8aXxUPNTNXaGzyoMRvwOtXSyVqikc%3D&reserved=0)) and the following text: “I request permission for the open-access journal PLOS ONE to publish XXX under the Creative Commons Attribution License (CCAL) CC BY 4.0 ([http://creativecommons.org/licenses/by/4.0/](https://eur03.safelinks.protection.outlook.com/?url=http%3A%2F%2Fcreativecommons.org%2Flicenses%2Fby%2F4.0%2F&data=05%7C02%7CC.Ostler%40soton.ac.uk%7C7cad507318504b07d1d808dc703e0007%7C4a5378f929f44d3ebe89669d03ada9d8%7C0%7C0%7C638508658790346043%7CUnknown%7CTWFpbGZsb3d8eyJWIjoiMC4wLjAwMDAiLCJQIjoiV2luMzIiLCJBTiI6Ik1haWwiLCJXVCI6Mn0%3D%7C0%7C%7C%7C&sdata=tVJ0S1xyG%2F3ue2rw943T54RTkHwWdE%2Ftd03Jo8SGzQY%3D&reserved=0)). Please be aware that this license allows unrestricted use and distribution, even commercially, by third parties. Please reply and provide explicit written permission to publish XXX under a CC BY license and complete the attached form.” Please upload the completed Content Permission Form or other proof of granted permissions as an ""Other"" file with your submission.   In the figure caption of the copyrighted figure, please include the following text: “Reprinted from [ref] under a CC BY license, with permission from [name of publisher], original copyright [original copyright year].” b. If you are unable to obtain permission from the original copyright holder to publish these figures under the CC BY 4.0 license or if the copyright holder’s requirements are incompatible with the CC BY 4.0 license, please either i) remove the figure or ii) supply a replacement figure that complies with the CC BY 4.0 license. Please check copyright information on all replacement figures and update the figure caption with source information. If applicable, please specify in the figure caption text when a figure is similar but not identical to the original image and is therefore for illustrative purposes only. | Written permission via email and the content permission form have been included in the re-submission as requested. |
| 4. | We note that Figure 2 in your submission contain [map/satellite] images which may be copyrighted. All PLOS content is published under the Creative Commons Attribution License (CC BY 4.0), which means that the manuscript, images, and Supporting Information files will be freely available online, and any third party is permitted to access, download, copy, distribute, and use these materials in any way, even commercially, with proper attribution. For these reasons, we cannot publish previously copyrighted maps or satellite images created using proprietary data, such as Google software (Google Maps, Street View, and Earth). For more information, see our copyright guidelines: [http://journals.plos.org/plosone/s/licenses-and-copyright](https://eur03.safelinks.protection.outlook.com/?url=http%3A%2F%2Fjournals.plos.org%2Fplosone%2Fs%2Flicenses-and-copyright&data=05%7C02%7CC.Ostler%40soton.ac.uk%7C7cad507318504b07d1d808dc703e0007%7C4a5378f929f44d3ebe89669d03ada9d8%7C0%7C0%7C638508658790352717%7CUnknown%7CTWFpbGZsb3d8eyJWIjoiMC4wLjAwMDAiLCJQIjoiV2luMzIiLCJBTiI6Ik1haWwiLCJXVCI6Mn0%3D%7C0%7C%7C%7C&sdata=%2FQkbousKO7%2FAzyXkBQATntWV0%2FSFuse3lv31FiUZBKE%3D&reserved=0).   We require you to either (1) present written permission from the copyright holder to publish these figures specifically under the CC BY 4.0 license, or (2) remove the figures from your submission:   a. You may seek permission from the original copyright holder of Figure 2 to publish the content specifically under the CC BY 4.0 license.     We recommend that you contact the original copyright holder with the Content Permission Form ([http://journals.plos.org/plosone/s/file?id=7c09/content-permission-form.pdf](https://eur03.safelinks.protection.outlook.com/?url=http%3A%2F%2Fjournals.plos.org%2Fplosone%2Fs%2Ffile%3Fid%3D7c09%2Fcontent-permission-form.pdf&data=05%7C02%7CC.Ostler%40soton.ac.uk%7C7cad507318504b07d1d808dc703e0007%7C4a5378f929f44d3ebe89669d03ada9d8%7C0%7C0%7C638508658790359506%7CUnknown%7CTWFpbGZsb3d8eyJWIjoiMC4wLjAwMDAiLCJQIjoiV2luMzIiLCJBTiI6Ik1haWwiLCJXVCI6Mn0%3D%7C0%7C%7C%7C&sdata=ThIK1Lcb7j0VNRLypWzQzI2bhf0nUAGOvJybCATt%2BiI%3D&reserved=0)) and the following text: “I request permission for the open-access journal PLOS ONE to publish XXX under the Creative Commons Attribution License (CCAL) CC BY 4.0 ([http://creativecommons.org/licenses/by/4.0/](https://eur03.safelinks.protection.outlook.com/?url=http%3A%2F%2Fcreativecommons.org%2Flicenses%2Fby%2F4.0%2F&data=05%7C02%7CC.Ostler%40soton.ac.uk%7C7cad507318504b07d1d808dc703e0007%7C4a5378f929f44d3ebe89669d03ada9d8%7C0%7C0%7C638508658790366921%7CUnknown%7CTWFpbGZsb3d8eyJWIjoiMC4wLjAwMDAiLCJQIjoiV2luMzIiLCJBTiI6Ik1haWwiLCJXVCI6Mn0%3D%7C0%7C%7C%7C&sdata=6IgNhYq8SaSztJBkgWJW%2BZsImNtb2x8SfHkTZSxci3Y%3D&reserved=0)). Please be aware that this license allows unrestricted use and distribution, even commercially, by third parties. Please reply and provide explicit written permission to publish XXX under a CC BY license and complete the attached form.”   Please upload the completed Content Permission Form or other proof of granted permissions as an ""Other"" file with your submission.   In the figure caption of the copyrighted figure, please include the following text: “Reprinted from [ref] under a CC BY license, with permission from [name of publisher], original copyright [original copyright year].”   b. If you are unable to obtain permission from the original copyright holder to publish these figures under the CC BY 4.0 license or if the copyright holder’s requirements are incompatible with the CC BY 4.0 license, please either i) remove the figure or ii) supply a replacement figure that complies with the CC BY 4.0 license. Please check copyright information on all replacement figures and update the figure caption with source information. If applicable, please specify in the figure caption text when a figure is similar but not identical to the original image and is therefore for illustrative purposes only. The following resources for replacing copyrighted map figures may be helpful:   USGS National Map Viewer (public domain): [http://viewer.nationalmap.gov/viewer/](https://eur03.safelinks.protection.outlook.com/?url=http%3A%2F%2Fviewer.nationalmap.gov%2Fviewer%2F&data=05%7C02%7CC.Ostler%40soton.ac.uk%7C7cad507318504b07d1d808dc703e0007%7C4a5378f929f44d3ebe89669d03ada9d8%7C0%7C0%7C638508658790374470%7CUnknown%7CTWFpbGZsb3d8eyJWIjoiMC4wLjAwMDAiLCJQIjoiV2luMzIiLCJBTiI6Ik1haWwiLCJXVCI6Mn0%3D%7C0%7C%7C%7C&sdata=RnlOK3%2F2S2Km04ROzaEjtM8tNXbhjIP6X1NALLXolTM%3D&reserved=0) The Gateway to Astronaut Photography of Earth (public domain): [http://eol.jsc.nasa.gov/sseop/clickmap/](https://eur03.safelinks.protection.outlook.com/?url=http%3A%2F%2Feol.jsc.nasa.gov%2Fsseop%2Fclickmap%2F&data=05%7C02%7CC.Ostler%40soton.ac.uk%7C7cad507318504b07d1d808dc703e0007%7C4a5378f929f44d3ebe89669d03ada9d8%7C0%7C0%7C638508658790381431%7CUnknown%7CTWFpbGZsb3d8eyJWIjoiMC4wLjAwMDAiLCJQIjoiV2luMzIiLCJBTiI6Ik1haWwiLCJXVCI6Mn0%3D%7C0%7C%7C%7C&sdata=dUakshjlTd30DdIorCLlj2P6J1ckLLCvX%2BxqRMVPENk%3D&reserved=0) Maps at the CIA (public domain): [https://www.cia.gov/library/publications/the-world-factbook/index.html](https://eur03.safelinks.protection.outlook.com/?url=https%3A%2F%2Fwww.cia.gov%2Flibrary%2Fpublications%2Fthe-world-factbook%2Findex.html&data=05%7C02%7CC.Ostler%40soton.ac.uk%7C7cad507318504b07d1d808dc703e0007%7C4a5378f929f44d3ebe89669d03ada9d8%7C0%7C0%7C638508658790388448%7CUnknown%7CTWFpbGZsb3d8eyJWIjoiMC4wLjAwMDAiLCJQIjoiV2luMzIiLCJBTiI6Ik1haWwiLCJXVCI6Mn0%3D%7C0%7C%7C%7C&sdata=GcKgHjD06dUFgtnyoHoLmTdTEHyYc%2Fy5e6ll9vE4OhY%3D&reserved=0) and [https://www.cia.gov/library/publications/cia-maps-publications/index.html](https://eur03.safelinks.protection.outlook.com/?url=https%3A%2F%2Fwww.cia.gov%2Flibrary%2Fpublications%2Fcia-maps-publications%2Findex.html&data=05%7C02%7CC.Ostler%40soton.ac.uk%7C7cad507318504b07d1d808dc703e0007%7C4a5378f929f44d3ebe89669d03ada9d8%7C0%7C0%7C638508658790395291%7CUnknown%7CTWFpbGZsb3d8eyJWIjoiMC4wLjAwMDAiLCJQIjoiV2luMzIiLCJBTiI6Ik1haWwiLCJXVCI6Mn0%3D%7C0%7C%7C%7C&sdata=WwiwY8892bzO6yXQo4FCKkCKZv2LtPVlaEjbNQ6NdEg%3D&reserved=0) NASA Earth Observatory (public domain): [http://earthobservatory.nasa.gov/](https://eur03.safelinks.protection.outlook.com/?url=http%3A%2F%2Fearthobservatory.nasa.gov%2F&data=05%7C02%7CC.Ostler%40soton.ac.uk%7C7cad507318504b07d1d808dc703e0007%7C4a5378f929f44d3ebe89669d03ada9d8%7C0%7C0%7C638508658790403825%7CUnknown%7CTWFpbGZsb3d8eyJWIjoiMC4wLjAwMDAiLCJQIjoiV2luMzIiLCJBTiI6Ik1haWwiLCJXVCI6Mn0%3D%7C0%7C%7C%7C&sdata=cvdKe2qIJ7EaA8cgXn2rVxcnRreY%2BBBS1O4AkuEZ7Vo%3D&reserved=0) Landsat: [http://landsat.visibleearth.nasa.gov/](https://eur03.safelinks.protection.outlook.com/?url=http%3A%2F%2Flandsat.visibleearth.nasa.gov%2F&data=05%7C02%7CC.Ostler%40soton.ac.uk%7C7cad507318504b07d1d808dc703e0007%7C4a5378f929f44d3ebe89669d03ada9d8%7C0%7C0%7C638508658790412655%7CUnknown%7CTWFpbGZsb3d8eyJWIjoiMC4wLjAwMDAiLCJQIjoiV2luMzIiLCJBTiI6Ik1haWwiLCJXVCI6Mn0%3D%7C0%7C%7C%7C&sdata=hUWBzfS1OHXSKs5QriysPjMUuxoZ%2F0x3lkzui2Yof1c%3D&reserved=0) USGS EROS (Earth Resources Observatory and Science (EROS) Center) (public domain): [http://eros.usgs.gov/#](https://eur03.safelinks.protection.outlook.com/?url=http%3A%2F%2Feros.usgs.gov%2F%23&data=05%7C02%7CC.Ostler%40soton.ac.uk%7C7cad507318504b07d1d808dc703e0007%7C4a5378f929f44d3ebe89669d03ada9d8%7C0%7C0%7C638508658790423280%7CUnknown%7CTWFpbGZsb3d8eyJWIjoiMC4wLjAwMDAiLCJQIjoiV2luMzIiLCJBTiI6Ik1haWwiLCJXVCI6Mn0%3D%7C0%7C%7C%7C&sdata=jfieO%2ByW%2BeCmAu7utC%2FQqiUySmPyTngiare5W8iOAPs%3D&reserved=0) Natural Earth (public domain): [http://www.naturalearthdata.com/](https://eur03.safelinks.protection.outlook.com/?url=http%3A%2F%2Fwww.naturalearthdata.com%2F&data=05%7C02%7CC.Ostler%40soton.ac.uk%7C7cad507318504b07d1d808dc703e0007%7C4a5378f929f44d3ebe89669d03ada9d8%7C0%7C0%7C638508658790434372%7CUnknown%7CTWFpbGZsb3d8eyJWIjoiMC4wLjAwMDAiLCJQIjoiV2luMzIiLCJBTiI6Ik1haWwiLCJXVCI6Mn0%3D%7C0%7C%7C%7C&sdata=4UW1wnreuRJlOhlqGPOQ6iYps1q1q16YubYgeoz2CNI%3D&reserved=0) | A replacement figure has been provided which complies with the CC BY 4.0 license as requested. |
| 5. | Please upload a copy of Figure 4, to which you refer in your text on page 34. If the figure is no longer to be included as part of the submission please remove all reference to it within the text. | Figure four on paged 34 has been corrected to figure three. This may not have been updated following removal of a figure to the supplementary information. Apologies for any confusion. |
| 6. | Please include captions for your Supporting Information files at the end of your manuscript, and update any in-text citations to match accordingly. Please see our Supporting Information guidelines for more information: [http://journals.plos.org/plosone/s/supporting-information](https://eur03.safelinks.protection.outlook.com/?url=http%3A%2F%2Fjournals.plos.org%2Fplosone%2Fs%2Fsupporting-information&data=05%7C02%7CC.Ostler%40soton.ac.uk%7C7cad507318504b07d1d808dc703e0007%7C4a5378f929f44d3ebe89669d03ada9d8%7C0%7C0%7C638508658790444998%7CUnknown%7CTWFpbGZsb3d8eyJWIjoiMC4wLjAwMDAiLCJQIjoiV2luMzIiLCJBTiI6Ik1haWwiLCJXVCI6Mn0%3D%7C0%7C%7C%7C&sdata=Sk11B974%2BFN%2FGPwrjMwcS%2BFzUz9yrt52QhYg9cOfQPg%3D&reserved=0). | A caption has been included at the end of the manuscript and in text citations have been updated to match accordingly. |
| 7. | Please review your reference list to ensure that it is complete and correct. If you have cited papers that have been retracted, please include the rationale for doing so in the manuscript text, or remove these references and replace them with relevant current references. Any changes to the reference list should be mentioned in the rebuttal letter that accompanies your revised manuscript. If you need to cite a retracted article, indicate the article’s retracted status in the References list and also include a citation and full reference for the retraction notice | The reference list has been reviewed and updated as requested. |
| **Reviewer 1** |  |  |
| Line 145 | The authors searched for 2011 and early 2023. Can this data be specified so that when others reproduce your search, they will get the same results? | Thank you for raising this. The month of the search has now been included. |
| 145-155 | Initially, the authors state that two researchers conducted screening. Why did only one author conduct the full-text screening? | Apologies for the confusion here. The text has been altered to clearly state that two reviewers undertook title and abstract screening, and a single reviewer undertook full text screening. |
| Table 4 | Table 4: How the pre-existing model was developed is unclear. | Thank you for highlighting this lack of clarity. The pre-existing model was developed in a qualitative study undertaken by the authors and listed as reference 20. This section has been rewritten to link to this paper and provide summary information about the study more clearly. This includes information about how the model was developed and how it was used to create the a priori framework. Please see lines 192 to 196) |
|  | What sample was used to develop this framework? | See comment above and lines 192 to 196. |
|  | How was the model developed? | See comment above and lines 192 to 196. |
| Line 228 | the authors use the PRISMA diagram. Please state that you have used the PRISMA guidelines in the method section. | This statement has been added to the research design section of the methods (see line 115). |
| Results | Can the different studies be compared to each other? Is the confirmability of the various studies sufficiently large? | Thank you for raising this issue. We have included additional text which attempts to discuss this concern in the final paragraphs of the discussion section and the limitations section (Lines 596-612 of track changed version). We have used the book you suggested below and based our additions on the critical reflections about qualitative literature and trustworthiness. We have tried to position these issues using the terminology set out by Lincoln and Guba i.e., credibility, transferability, dependability and confirmability. Thank you for this information. |
| Discussion and conclusion | The authors present the ECLIPSE model based on a literature review. Can the results be generalised, given the variety of included studies? | Thank you for raising this issue. We have included additional text which attempts to discuss this concern in the final paragraphs of the discussion section and the limitations section (Lines 596-612 of track changed version). We have used the book you suggested below and based our additions on the critical reflections about qualitative literature and trustworthiness. We have tried to position these issues using the terminology set out by Lincoln and Guba i.e., credibility, transferability, dependability and confirmability. Thank you for this information. |
|  | The authors state that most of the factors included in the framework intertwine. Does the framework then cover the patient perspectives sufficiently? Should the framework be adapted also to include the interaction between the different domains? How does this influence the credibility of the results? | In the discussion section we have highlighted that the ECLIPSE model represents a conceptual model. We also state that the data we have synthesised suggests there are relationships between these different concepts, and we highlight some examples. We have not attempted to go further and describe the nature of the relationships in this paper. We summarise that further research would be required to do this, perhaps in an attempt to develop theory related to recovery following prosthetic rehabilitation. However, this is outside the scope of this current paper. |
|  | Overall, the authors should be cautious concerning statements about applying the framework. | Thank you for this comment. The discussion and conclusion sections have been reviewed to soften the language used when discussing application of the framework to reflect this caution.  The limitations section has also been reviewed and developed in light of previous comments about trustworthiness. |
|  | The methods, results and discussion sections have been evaluated by applying Truthworthiness from O’Reilly, M., & Kiyimba, N. (2015). Advanced qualitative research. (Vols. 1-0). SAGE Publications Ltd, <https://doi.org/10.4135/9781529622782> | Thank you for this useful reference. |
| **Reviewer 2** |  |  |
|  | This article develops a qualitative framework to define and structure relevant outcomes for patients after lower limb amputation. This article builds on the authors' preliminary work on a framework that was initially developed with few (37) and only UK patients. In this paper, an extended framework is derived from a systematic literature review and a synthesis of the qualitative findings from the relevant publications. This new model is based on 40 studies involving 539 patients. This makes the database much broader and more representative (subjects from 15 countries), although the authors point out the (too) few studies from low-income countries, which could introduce a systematic bias. On the basis of these data, the authors have extended their original model.  The work is highly relevant, as the definition of outcomes and then their measurement is of great importance for health care. The inclusion of the perspective of those affected - in this case people with amputations - is particularly important, as only they can assess the subsequent relevance for their everyday life and participation. This perspective also determines the qualitative approach chosen by the authors. | Thank you for this summary and feedback. |
|  | The initial problem definition and the systematics of the study are well presented by the authors. The methodology is appropriate to the problem and the main methodological steps and interim results are well described. The assessment of the quality of the included studies is also interesting, although the exact impact of the assessment on the further analysis/synthesis is not entirely clear. | Thank you for raising this point. As stated in the methods the critical appraisal process was used to give context to the findings presented in the synthesis, and comment on the quality of the overall sample. In this section we highlight some of the issues surrounding qualitative appraisal within qualitative syntheses.  To clarify these issues further and justify the extent to which we have incorporated the critical appraisal findings, additional text has been added to the end of the discussion section, prior to the limitations, setting out issues with critical appraisal. We have also added text linking back to the key issues identified in this review’s critical appraisal and their implications for the findings. |
|  | The actual synthesis consists of two parts: i) the collection of evidence for the existing framework and its domains and ii) the addition of additional sub-themes to the framework. Regarding i), the authors found sufficient evidence to support the framework already developed and therefore further development seems reasonable. Regarding 2), while only minor adjustments were made to some domains (2 and 4), domain 1 was expanded to include the relevant roles of those affected and domain 5 was expanded to include additional aspects of subjective coping and acceptance of 'my new normal'. Domain 3 was split into two old and one new sub-theme. However, sub-theme 3.3 (My prosthesis enables me to participate) does not seem to be clearly distinct from domains 1 and 2. The description and quotes seem to focus more on subjective trust in the prosthesis and the steps involved in building trust. | Thank you for highlighting this lack of clarity with Domain 3. We have attempted to clarify how the theme was previously defined and why it has been respecified following the best fit framework analysis. In the original conceptual model, domain three focused on comfort and ease of use. Ease of use included the burden of fluctuating socket fit, suspension mechanisms and weight of the prosthesis, but participants did not discuss prosthetic componentry. The review and best fit framework synthesis identified data focusing on componentry and how important the right components were to enable participation. Subtheme three tries to describe this phenomenon. Hopefully our additional text has helped to clarify this. |
|  | Overall, the existing framework becomes more comprehensive and differentiated due to the broader database. However, when describing the new aspects, the previous framework should be more clearly delineated, otherwise the additions are not comprehensible and their relevance is not clear | We have attempted to add text throughout the results section which more clearly describes the previous framework and improves comprehensibility of the new additions. The changes are highlighted in the tables linked to each domain, but we hope the additional text adds clarity. |
